# Analysis of YouTube Videos on Pregnant COVID-19 Patients During the Pandemic Period

**DOI:** 10.7759/cureus.29934

**Published:** 2022-10-05

**Authors:** Ayhan Atigan

**Affiliations:** 1 Obstetrics and Gynaecology, Karabuk University, Karabük, TUR

**Keywords:** pandemic, pregnancy, youtube, coronavirus, sars-cov-2, covid-19 retro

## Abstract

Objective: Pregnant women often refer to YouTube videos when they are worried about themselves and/or their baby. This study aims to evaluate COVID-19 and pregnancy-related content on YouTube, the platform that comes to mind first when social media is mentioned.

Methods: YouTube videos were evaluated between September 7-10, 2022. “COVID-19 pregnancy” videos with more than 100.000 views were included in the study. The content and technical data of 45 videos were recorded. The videos were scored using the DISCERN score, Video Power Index (VPI), and Global Quality Scale (GQS).

Results: Of the 45 videos with a mean duration of 432 seconds, 32 (71.1%) of them originated in the USA, 38 (84.4%) of them were presented by healthcare workers, and 36 (80.0%) of them recommended vaccination. Accounts producing the videos had a mean of 3,037,619 subscribers. The videos we analyzed were viewed a mean of 522836 times. These videos had 9287 likes and 1891 comments. The DISCERN, VPI, and GQS mean scores of the videos were 32.36, 74.76, and 3.82, respectively.

Conclusion: In order to correctly inform society about health problems, healthcare workers must make presentations on YouTube with a controlled mechanism. There is confusion about information on the internet, and people must acquire information selectively.

## Introduction

A pandemic was declared in 2020 with the detection of severe acute respiratory syndrome coronavirus 2 (SARS-CoV-2) [1]. SARS-CoV-2 is a single-stranded RNA virus with a genome that encodes a variety of proteins [2]. Alpha, beta, gamma, and delta variants have been reported according to their genomic structures [3]; the symptoms may vary depending on the variant acquired. Common symptoms of the novel coronavirus disease 2019 (COVID-19) include anosmia, fever, cough, myalgia, headache, vomiting, and diarrhea [4]. Even though strict rules such as mask use, remote work, social distance, and even isolation were implemented to prevent the spread of COVID-19, cases, unfortunately, increased rapidly. Due to the increasing number of cases, global lockdown and quarantine protocols were implemented which had several negative effects on the social, psychological, and business life of people. Almost a year later, a safe and effective vaccine became available [3].

Pregnancy carries a high risk of morbidity and mortality in case of infection with the COVID-19 virus [4,5]. Pneumonia is one of the most common causes of non-obstetric infections and indirect maternal deaths. One in four women who develop pneumonia requires hospitalization for respiratory support [6]. According to a study based on the literature, it was stated that pneumonia increased premature rupture of membranes (PROM), preterm labor (PTL), intrauterine fetal death (IUFD), intrauterine growth restriction (IUGR), and neonatal death [6]. It is easily transmitted in the family. However, no mother-to-baby transmission was observed in the womb. Although the placental infection cannot be demonstrated, perinatal transmission may occur rarely [4]. There are congenital birth defects that have been noticed or not yet recognized due to COVID-19 [7].

Youtube, which is one of the most used social platforms in daily life due to its visual content, has millions of members. Since it is a platform where everyone can upload videos without undergoing a detailed review, patients, healthcare professionals, and ordinary people benefit from the videos, even if there is doubt about the reliability of the content [8]. Pregnant women conduct internet research on almost any topic that comes to mind and organize quickly through groups. The amount of time spent at home and on the internet has increased significantly, particularly since the global lockdown. We examined YouTube for COVID-19-related pregnancy videos which are a virtual reflection of society because people from all walks of life both produce and watch video content. We aimed to emphasize the priority of pregnant women in the pandemic as well as discuss what we learned for the effective management of subsequent pandemics.

## Materials and methods

Data collection

Study data were collected from YouTube between September 7-10, 2022. A search was made by typing "COVID-19 pregnancy" in the YouTube search bar in Google Chrome incognito tabs. Videos were sorted by the number of views. The data were collected and interpreted by the author. Videos with more than 100,000 views were included in the study. Non-English and irrelevant videos with less than 100,000 views were not included in the study.

Videos in the English language with over 100,000 views were analyzed in detail. The following parameters were noted: video uploader, country, content, duration, total views, likes, dislikes, number of subscribers, and number of comments. The videos were checked to see if the following content was mentioned: primary prevention methods, vaccination, treatment, the problems it brings to health workers, its effects on social, psychological, and business life, its effects on breastfeeding and pregnancy outcomes, causes of miscarriage, maternal death, anomaly, and variants.

Ethics committee approval was not required for this study as it did not present any personal data that is easily accessible to everyone.

Data analysis

Video quality parameters were determined. The Video Power Index (VPI) score was computed by using the formula [likes/(likes+dislikes)] x 100 [9]. Moreover, the DISCERN and Global Quality Scale (GQS) were calculated. DISCERN is a rating scale with three sections and a total of 16 questions. In this rating scale, in which good scores demonstrate quality, the scores for each question range from one to five points [10]. Similarly, GQS is scored from one (poor quality) to five (excellent quality) [11].

Statistical analysis

Statistical analyses were performed using SPSS, version 21 (IBM Corp., Armonk, NY). Continuous variables were expressed as mean±standard deviation, and categorical variables were expressed as percentages.

## Results

Analysis of video scores is presented in Table 1. The videos had a mean duration of seven minutes and 12 seconds (±8.29), 522,836 (±1,236,404) total views, and 9,287 (±28,644) mean likes. The mean values of the video quality scales were VPI 74.76±33.96, GQS 3.82±1.11, and DISCERN 32.36±9.22. Thirty-eight healthcare workers (84.4%), three patients (6.7%), four no-voice (8.9%) video presenters emphasized protection in 32 (71.1%) videos, COVID management in 11 (24.4%), and health policies in two (4.4%) videos. The distribution of YouTubers by country was the following: 32 (71.1%) videos were from the USA, six (13.3%) from the UK, and seven (15.6%) from other countries. The mean number of comments on the videos was 1,891±3,775. The mean subscriber count of YouTubers was 3,037,619±3,937,342.

**Table 1:** Analysis of video scores VPI: Video Power Index; GQS: Global Quality Scale

Features		Mean	Std Deviation	Median		Minimum	Maximum
Video duration, minutes		7.12	8.29	3.20		0.30	41.18
Total views		522,836	1,236,404	207,967		100,971	8,250,482
Likes		9,287	28,644	1,845		1	154,406
VPI		74.76	33.96	95.21		9.87	100.00
GQS		3.82	1.11	4.00		1.00	5.00
DISCERN		32.36	9.22	32.00		18.00	55.00
Subscriber counts		3,037,619	3,937,342	1,210,000		1,240	16,100,000
Comment counts		1,891	3,775	318		0	21,573
		N			%		
Country group	USA	32			71.1%		
	UK	6			13.3%		
	Other countries	7			15.6%		
Video presenter	Health Worker	38			84.4%		
	Patient	3			6.7%		
	No voice	4			8.9%		
Emphasized	Protection	32			71.1%		
	Covid Management	11			24.4%		
	Pandemic Health Policies	2			4.4%		

The featured topics are presented in Table 2. Vaccination was recommended directly in 36 (80.0%) videos, and non-vaccine prevention methods were explained in only 10 (22.2%) videos. Symptomatic treatments for COVID-19 were explained in 10 (22.2%) videos, and the problems experienced by healthcare personnel were explained in two (4.4%) videos. Poor pregnancy outcomes such as fetal growth restriction and preterm delivery were discussed in 32 (71.1%) videos. Things to consider during breastfeeding of COVID-19 were explained in 16 (35.6%) videos. The psychological effects as well as the effect on work and social life were discussed in 12 (26.7%) videos. COVID-19 symptoms were described in 11 (24.4%) videos. COVID-19 has been linked to miscarriage in 16 (35.6%) videos and maternal mortality in 10 (22.2%) videos. In only three (6.7%) videos, fetal anomaly status was mentioned for both COVID-19 and vaccines, and it was stated that it did not cause an anomaly. Variants were mentioned in only two videos for SARS-CoV-2.

**Table 2:** The topics mentioned in the videos

The mentioned topics	Yes n (%)	No n (%)
Non-vaccine protection	10 (22.2%)	35 (77.8%)
Vaccination	36 (80.0%)	9 (20.0%)
Specific treatment	10 (22.2%)	35 (77.8%)
Problems faced by healthcare professionals	2 (4.4%)	43 (95.6%)
Effect on pregnancy outcomes	32 (71.1%)	13 (28.9%)
Effect on breastfeeding	16 (35.6%)	29 (64.4%)
Impact on psychology, work and social life	12 (26.7%)	33 (73.3%)
Symptoms	11 (24.4%)	34 (75.6%)
Miscarriages	16 (35.6%)	29 (64.4%)
Maternal mortality	10 (22.2%)	35 (77.8%)
Anomaly	3 (6.7%)	42 (93.3%)
Variant	2 (4.4%)	43 (95.6%)

## Discussion

"That which does not kill us, makes us stronger," said Nietzsche. Although the thought that society might perish during the pandemic comes to mind, we are getting out of this situation by getting stronger and increasing our knowledge. In our study, we evaluated the most watched YouTube videos during the pandemic lockdown. There are around three hundred thousand studies associated with COVID-19 in PubMed. The experience of healthcare workers has greatly increased. This painful experience means hard work, experiencing the illness yourself, and suffering the loss of loved ones. While the healthcare community has been working hard, most employees have stopped working. Schools were on holiday and children could not even go out on the streets to play. Even medical school students could not complete their clinical rotations properly [12]. A study on the levels of burnout in healthcare workers during COVID-19 reported that it caused psychological problems, and employees had to leave their family homes and required significant lifestyle changes [13]. Although these situations are partially explained in YouTube videos, we have all experienced them by living.

In a COVID-19 YouTube study, it was stated that videos can create an atmosphere of fear and chaos by mentioning death too much [14]. In the videos in our study, maternal death and miscarriage possibilities were mentioned to a considerable extent. COVID-19 can cause mortality due to conditions such as breathing difficulties and pneumonia. The virus can cause a cytokine storm in people who are sick [2]. Specific treatment drugs are also being developed as a result of a better understanding of the pathogenesis. It is aimed to strengthen the immunity of the patient and reduce the production of inflammatory cytokines [2]. There are suspicions that hydroxychloroquine, one of the drugs used in the management of COVID-19, may cause death [7]. In the majority of the videos, it has been reported that preterm birth and fetal growth restriction can be seen as the bad effects of having COVID-19 during pregnancy. Watching these videos can be a nightmare for anxious pregnant women. It has been emphasized in the literature that there may be fetal distress and premature birth due to COVID-19 and its associated pneumonia [4,6]. Fortunately, antibodies are present in milk, although SARS-CoV-2 was not detected in milk during lactation. Since breastfeeding requires close contact, isolation rules should be strictly followed, especially during active infection [15]. Viral effects of SARS-CoV-2 in the early embryonic period may cause congenital defects. Neurodevelopmental complications that can be caused by high fever have not yet been clarified. There are doubts that favipiravir, which also has a place in the management of COVID-19, causes congenital defects [7]. Information about congenital anomalies is provided in only three videos. However, it is especially emphasized that COVID-19 does not have a congenital anomaly effect in all three videos.

VPI was found to be lower in our study as compared to previous studies that were calculated similarly [9,16,17]. The number of likes was higher in our study. However, video duration, number of views, GQS and DISCERN scores were similar to previous studies [9,16,17]. The videos in our study had more comments. Of course, one of the reasons for more likes and comments in our study may be the mystical aspect of pregnancy and COVID-19 [9,16,17]. The main emphasis of our study was that the videos were of USA origin and were presented by healthcare workers on the subject of protection and immunity. In other words, it can be said that the healthcare professionals in the USA care about vaccination for COVID-19 and this has resonated widely. In the current study, where we scanned the most watched videos, one reason for the low expression of COVID-19 symptoms during pregnancy may be that the symptoms are sufficiently known. What is interesting is that although almost all of the videos talk about prevention and COVID-19 management, SARS-CoV-2 variants are barely mentioned. Perhaps the fact that the variants have not received adequate attention is the reason the pandemic has lasted so long.

There are some limitations in our study. Unfortunately, data such as the number of views, comments, and likes of YouTube videos keep changing over time. Since the videos were not grouped, they could not be compared. The videos were analyzed using descriptive epidemiological research methods. Another limitation is that the videos with less than a hundred thousand views were not reviewed.

## Conclusions

In this modern age we live in, Youtube, where countless videos are uploaded and millions of videos are watched every day, has a huge social impact. Although some videos cause disinformation, the most practical way of accessing the right information is through the internet and especially on YouTube. Just as COVID-19 articles are increasingly cited over the years, these videos will be a source of information for new epidemics that may occur in the following years. Pregnant women seek answers to their questions online and often when they cannot find the relevant information, they quickly organize and discuss their situation on forum pages. Due to this reason, it may be beneficial to present content on YouTube which contains the correct information provided by healthcare professionals and especially obstetricians that address the concerns of pregnant women.
